# Effects of Diagnostic Labels for Students With Learning Problems on Teachers’ Stereotypes and Performance Expectations

**DOI:** 10.1177/00222194251315187

**Published:** 2025-03-16

**Authors:** Linda Kashikar, Timo Lüke, Michael Grosche

**Affiliations:** 1 University of Wuppertal, Germany; 2 University of Kassel, Germany

**Keywords:** learning disabilities, label, teachers’ expectations, stereotypes, general education, special education

## Abstract

Labeling students with learning problems may change teachers’ evaluations of them. Our study examined whether the “Special Learning Needs” (SLN) and “Learning Disability” (LD) labels influenced teachers’ beliefs that the diagnosis was correct and activated a low-competence stereotype. We examined whether this stereotype lowered teachers’ performance expectations and teaching intentions. A sample of 413 German general and special education teachers were randomly assigned to the control (no label) or one of the experimental groups (SLN/LD label). All teachers read the description of a fictitious student with learning problems. Only in the experimental groups was the student labeled with an LD or SLN. Results showed that both labels increased teachers’ acceptance of the diagnosis as accurate. However, the labels did not change teachers’ stereotypes of the fictitious student. The LD label lowered some of teachers’ long-term performance expectations, resulting in more track recommendations to a special school. Unexpectedly, the SLN label increased the intention to foster the student’s academic performance. Some performance expectations of special education teachers were lower than those of general education teachers, which did not manifest in different teaching intentions. The findings are discussed in the context of the Dilemma of Difference.

Three teachers bullied my son. He was diagnosed with Special Learning Needs, and they thought he would not be able to achieve anything in the future. We got him out of the special education system, and now, he can graduate from college successfully. One must have a long breath as parents, but it is worth fighting.

This quote from a mother in a parents’ group reflects the problem often assumed on a theoretical level that the diagnosis of special educational needs can stigmatize these students. This phenomenon is known as the “Dilemma of Difference” (Norwich, 2009). It means that the necessary resources provided to support these students are linked to an official diagnosis of special educational needs, which may result in stigmatization.

A recent meta-analysis showed that labeling students with diagnoses (primarily medical and special educational needs) worsened teachers’ evaluations of these students (Franz et al., 2023). A theoretical framework postulated that diagnostic labels were linked to stereotypes, leading to the stigmatization of individuals (Link & Phelan, 2001), which is also assumed for special education labels (Ho, 2004).

The relation between adverse label effects and stereotypes in special education has been discussed theoretically; this study empirically examined the presumed connection between labels, their link to stereotypes, and teachers’ performance expectations of labeled students. Specifically, we investigated whether labeling students with learning problems with an official diagnosis affected teachers’ judgments of these students. First, we examined whether the labels increased teachers’ belief that the diagnosis was accurate. Second, we investigated whether this changed teachers’ stereotypes and related emotions. Third, we examined whether the labels’ link to stereotypes lowered teachers’ performance expectations of labeled students and changed their teaching intentions. In addition, we investigated whether these judgments differed between general and special education teachers.

Our theoretical model for this study is depicted in Figure 1 and can be summarized as follows: According to labeling theory (Link & Phelan, 2001), the special education labels “Learning Disability” and “Special Learning Needs” link labeled students to a negative group stereotype. The group stereotype of students with learning problems has previously been measured using the stereotype content model (Fiske et al., 2002), showing that teachers perceived this group of students as low in academic competence (Krischler et al., 2018). Low perceived competence resulted in low-performance expectations (Krischler & Pit-ten Cate, 2019), which manifested in teachers’ lower behavior intentions following the expectation effect model (Johnston et al., 2019). However, it is not explicitly mentioned in labeling theory, but based on our previous research (Kashikar et al., 2023, 2024), we postulate that diagnostic labels are only linked to the negative stereotype of low academic competence, resulting in lower performance expectations when perceived, actively remembered, and accepted as accurate. In the following sections, we will refer to our theoretical model as we explain every step in more detail.

**Figure 1.**
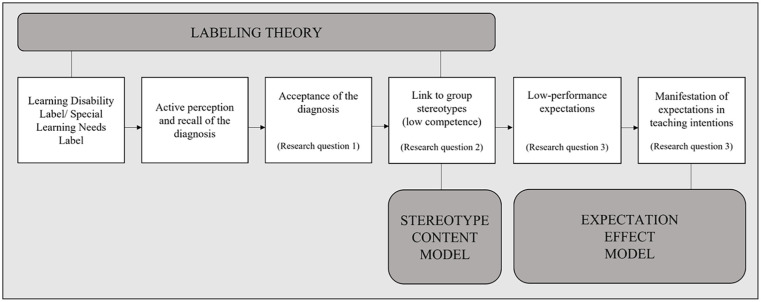
Theoretical Model of this Study Explaining How Labels Are Linked to Stereotypes and Performance Expectations.

## Ableism in Education, Labeling, Stereotypes, and Stigma

Ableism is broadly defined as “stereotyping, prejudice, discrimination, and social oppression toward people with disabilities” (Bogart & Dunn, 2019, p. 651). In schools, Brantlinger (2006) described ableism as follows: “Individuals and groups who fail to achieve dominant standards are identified (marked, labeled, branded) with stigmatizing names (e.g., failure, disabled, and at-risk) and sent to separated locations (special education rooms, low tracks, and vocational schools). These distinction-making processes create a binary of (dominant) insiders and (subordinate) outsiders” (p. 200).

The postulated connection between labeling and stigmatization of students in the school context can be explained by labeling theory (Goffman, 1963). A stigma is an “attribute that is deeply discrediting” and reduces someone “from a whole and usual person to a tainted, discounted one” (Goffman, 1963, p. 3). Link and Phelan (2001) specified the connection between labels and stigmatization in a revised four-component theory: First, people distinguish and label human differences. Second, labeled individuals are linked to undesirable characteristics, that is, negative stereotypes. Third, the label facilitates categorizing into “them” and “us.” Fourth, the labeled individuals experience status loss and discrimination, leading to poorer outcomes for labeled than nonlabeled individuals.

One focus of this study was the link between labels and stereotypes, as presented in Figure 1. Labeling occurs when individual differences are interpreted as relevant, meaningful, and significant. These labeled differences are often linked to stereotypes (Andersen et al., 2022). Stereotypes are defined as “people’s beliefs (cognitions) about an individual based on group membership” (Fiske, 2018, p. 4). Thus, if the group’s stereotype is negative, the label links a person to “a set of undesirable characteristics that form the stereotype” (Link & Phelan, 2001, p. 369). As positive stereotypes do not lead to stigmatization, only labels with negative connotations have the potential to stigmatize (Andersen et al., 2022; see Note 1).

In the school context, negative stereotypes are believed to lead to adverse effects of special education labels (Daley & Rappolt-Schlichtmann, 2018; Ho, 2004; Shifrer, 2013, 2016), which is usually explained with the stereotype content model (see Figure 1, Cuddy et al., 2007; Fiske et al., 2002). The model distinguishes the dimensions “competence” and “warmth,” which can be “low” or “high,” respectively, leading to four types of stereotypes and related emotions. Typically, the perceived competence of a group is predicted by its perceived status, and the perceived warmth is predicted by its perceived competition.

For example, “rich people” are viewed as high in status, predicting high perceived competence. They are also perceived as competitive and thus predict low perceived warmth. This combination is called envious stereotypes. “Disabled people” are perceived with high warmth but low competence, a combination called paternalistic stereotypes (Fiske et al., 2002).

Paternalistic stereotypes are believed to be expressed in paternalistic emotions, which are pity and sympathy (Cuddy et al., 2007). Both emotions are usually felt toward groups with negative outcomes for which they are not responsible (Fiske et al., 2002). Nevertheless, the two are distinctive emotions because “Pity combines sympathy with superiority” (Fiske et al., 2002, p. 899). Thus, higher sympathy than pity may indicate a more equivalent perceived status. Despite the negative connotation of the emotion pity, paternalistic emotions can evoke the willingness to provide help (Fiske et al., 2002). However, they do not necessarily lead to active help but may instead evoke avoidance and neglect as well (Cuddy et al., 2008). Thus, pity and sympathy as emotions related to stereotypes may manifest in different teaching behaviors. However, research about these theoretical assumptions for teachers’ stereotypes of students with learning problems is scarce.

Two studies examining teachers’ stereotypes of students with learning problems using the stereotype content model showed that teachers perceived these students as less competent than warm (Krischler et al., 2018; Krischler & Pit-ten Cate, 2019), indicating a tendency toward paternalistic stereotypes, which may elicit paternalistic emotions of pity and sympathy. This pattern can have both beneficial and detrimental effects on students. On one hand, students who perceive pity from their teachers in the event of academic failure are likely to attribute their teachers’ emotional response to the teachers’ belief that their failure is due to low ability (Georgiou et al., 2002; Graham, 1984). This may undermine their self-esteem. On the other hand, because of the possibility that paternalistic emotions evoke the willingness to help, it can be argued that they may have a protective effect on students (Krischler & Pit-ten Cate, 2019). However, unsolicited help may lower students’ self-esteem like pity does (Georgiou et al., 2002; Graham, 1984). To summarize, students with learning problems are likely to be viewed by teachers with paternalistic stereotypes, which may evoke teachers’ willingness to help but comes with a risk of a drop in students’ self-esteem.

How can the knowledge about labeling and stereotypes of students with learning problems be combined? If labeling students with learning problems with a special educational need diagnosis (i.e., a disability diagnosis) was linked to stereotypes described earlier, diagnostic labels for students with learning problems should result in lower perceived competence but higher warmth ratings for labeled students than identical but nonlabeled students. This assumption is supported by studies that showed that teachers expressed higher paternalistic emotions for students labeled with a LD than for similar nonlabeled students (Clark, 1997; Woodcock & Vialle, 2010, 2011).

As indicated in Figure 1 but not explicitly mentioned in the labeling theory, we assume that before the label is linked to stereotypes, teachers must first perceive and accept the diagnostic label as correct. If teachers do not perceive or doubt the diagnostic label, there would be no reason to link the label to group stereotypes, as the individual is not believed to belong to the group of students with a special needs diagnosis for learning problems. Indeed, our previous studies showed that the LD label makes it more likely that teachers believed that a fictitious student had a LD compared with an identical student without the assigned label (Kashikar et al., 2023, 2024).

## Diagnostic Labels for Students With Learning Problems and Teachers’ Expectations

Our theoretical model postulates that teachers have low-performance expectations of students they perceive as low in competence (see Figure 1). Studies showed that teachers’ ratings of students’ competence (but not warmth) were significantly related to teachers’ performance expectations of these students (Akifyeva & Alieva, 2018; Krischler & Pit-ten Cate, 2019). Also, a recent meta-analysis showed that academic evaluations, such as teachers’ performance expectations, were most negatively influenced by diagnostic labels for students compared with other assessments, like behavioral evaluations or attitudes toward students (Franz et al., 2023). Thus, this study also examined the effects of diagnostic labels for students with learning problems on teachers’ performance expectations.

Teachers’ performance expectations are “inferences that teachers make about the future behavior or academic achievement based on what they know about these students now” (Good, 1987, p. 32). They are essential because of their potential to influence students’ performance in a way that high expectations are associated with better performances and vice versa (Wang et al., 2018). The simplified expectation effect model explains how teachers’ expectations contribute to students’ academic performances: First, teachers develop expectations. Second, based on these expectations, teachers treat students differently. Third, students react to teachers’ behavior, and fourth, as a result, student outcomes improve or decline (Johnston et al., 2019; see Figure 1 displaying Steps 1 and 2 of the expectation effect model).

Studies showed that labeling students with learning problems with an official diagnosis lowered teachers’ performance expectations of these students compared with similar unlabeled students. These effects were observed in different contexts and for different diagnostic labels, such as the “LD” label (Kashikar et al., 2024; Shifrer, 2013; Whitley, 2010; Woodcock, 2014; Woodcock & Vialle, 2010, 2011), the “Specific Learning Difficulties” label (Woodcock, 2020; Woodcock & Vialle, 2016), and the “Dyslexia” label (Hornstra et al., 2010). Most of these studies focused on “classic” performance expectations, such as the likelihood of failing the next examination as a relatively short-term or the expected graduation level as a long-term expectation (i.e., Step 1 of the simplified expectation effect model). Because these expectations are likely to manifest in differential treatment of students (i.e., Step 2 of the simplified expectation effect model), we examined not only the influence of diagnostic labels of students with learning problems on teachers’ performance expectations but also their manifestation in behavioral intentions.

## Labels for Students With Learning Problems: Differential Effects on General and Special Education Teachers?

We examined differences in the label effects on general education teachers (GETs) and special education teachers (SETs), as both teaching professions are involved in inclusive education. Studies suggested that diagnostic labels for students did not influence GETs’ and SETs’ judgments of the labeled students differently (Bianco, 2005; Bianco & Leech, 2010; Franz et al., 2023; Kashikar et al., 2024).

However, regardless of the label, no previous study examined whether SETs and GETs hold different stereotypes of students with learning problems. Only careful and indirect conclusions can be drawn from studies that examined teachers’ performance expectations and perceived stability of students’ learning problems. However, these studies showed ambiguous results. Two studies demonstrated that GETs perceived learning problems as more stable and uncontrollable than SETs (Vlachou et al., 2014; Woolfson et al., 2007). If students’ learning problems were perceived as stable, this may stem from a low perceived competence. Contrarily, our studies showed that SETs had lower performance expectations, which may result from low perceived competence (Kashikar et al., 2023, 2024).

Due to these ambiguous results, this study aimed to examine whether the two teaching professions differed in their beliefs concerning the diagnosis, stereotypes, and performance expectations of students with learning problems and whether labeling students influenced these evaluations of the two teaching professions differently.

## Labels for Students With Learning Problems in This Study

This study was conducted in Germany. We examined two diagnostic labels for students with learning problems. The first label was the diagnosis “Lernbehinderung,” which translates to “LD”. The second was the “SLNs” label (“sonderpädagogischer Förder-/Unterstützungsbedarf im Lernen”). The German SLN expression emphasizes the need for support for the students. It is important to note that the two labels intended to describe the same group of students, but the SLN diagnosis officially replaced the LD diagnosis. The LD diagnosis was older, first introduced in 1972 as one of the ten different types of special schools, that is, the “school for the learning disabled” (“Schule für Lernbehinderte”; Heimlich, 2009; Powell, 2010). Due to a paradigm change to avoid a deficit-oriented and school-centered classification system, nine “categories of educational support” (“Fördersch-werpunkte”) were defined in 1994, one of them was “learning,” from which the current and official SLN diagnosis developed (Powell, 2010).

To summarize, students who were formerly labeled with an LD are currently officially diagnosed with SLN. However, although the term LD is criticized for its stigmatizing effect (Schröder, 2005), it is still informally used in everyday school life. Also, it is possible that the stereotypes linked to a former diagnostic nomenclature can be transferred to the newer diagnostic nomenclature (Werner & Abergel, 2018). For this reason, we examined the influence of both the SLN and LD labels on teachers’ judgments to identify possible differences in their effects on teachers’ evaluations.

It is also essential to describe the meaning of an LD and SLN in Germany, as the diagnostic criteria for special educational needs diagnoses differ between countries (Grünke & Cavendish, 2016). Both diagnoses describe students who show serious, global (i.e., in more than one subject), long-lasting learning and performance problems (Grünke & Grosche, 2014; Ministerium für Schule und Bildung des Landes Nordrhein-Westfalen, 2022). Thus, they differ from how a “specific LD” is defined by the *Diagnostic and Statistical Manual of Mental Disorders* (fifth ed.; *DSM-5*; American Psychiatric Association, 2013). However, the definitions of the German concepts of LD and SLN differ in their assumption about the cognitive functioning of the diagnosed students. Specifically, the official SLN diagnosis does not include deficits in cognitive functioning, although cognitive functioning is frequently tested in the diagnostic process (Lüttich, 2022). The criteria of an LD include deficits in cognitive functioning (Grünke & Grosche, 2014). Both diagnoses are also distinguished from an “intellectual disability” (American Psychiatric Association, 2013), as an LD/SLN primarily manifests in school problems. Contrarily, an intellectual disability will usually result in at least a minimal need for support in daily life (Häßler, 2011). The former LD and now SLN diagnoses were and are reevaluated at least yearly (Ministerium für Schule und Bildung des Landes Nordrhein-Westfalen, 2022).

## Research Questions and Hypotheses

Our study was an experiment in which teachers read a vignette describing a fictitious student with learning problems. We used a two-factorial design to test the main effects of the experimental group (vignette without label vs. vignette with an SLN label vs. vignette with an LD label) and the teaching profession (GETs vs. SETs) on teachers’ judgments of the fictitious student. We also examined the differential effects of the two labels on teachers’ judgments, that is, the interaction of the experimental groups and the teaching professions. Thus, our research questions were as follows:

1. **Research Question 1 (RQ1):** Do the experimental group, the teaching profession, and their interaction influence teachers’ beliefs that the diagnosis was correct?
2. **Research Question 2 (RQ2):** Do the experimental group, the teaching profession, and their interaction influence teachers’ stereotypical beliefs about the fictitious student?
3. **Research Question 3 (RQ3):** Do the experimental group, the teaching profession, and their interaction influence teachers’ expectations of the fictitious student’s future performances and their manifestation in teachers’ behavioral intentions regarding the performance requirements for the fictitious student?

As indicated in Figure 1, our previous studies showed that labels only unfolded their adverse effects on teachers’ expectations when teachers consciously remembered that the diagnosis was mentioned in the student’s description (Kashikar et al., 2023, 2024). For this reason, the adverse effects of the labels in this study were only hypothesized for teachers who remembered the respective diagnosis in the fictitious student’s description.

### Hypothesis 1

**H1a:** Teachers who remember that the fictitious student is labeled with an SLN/LD diagnosis believe more frequently that the diagnosis is correct.
**H1b:** SETs believe more frequently that the diagnosis is correct than GETs.

### Hypothesis 2

**H2a:** Teachers who remember that the fictitious student is labeled with an SLN/LD diagnosis have higher paternalistic stereotypes of and emotions for the student than teachers who do not remember the diagnosis or who read the student’s description without a diagnostic label.
**H2b:** SETs have more paternalistic stereotypes and emotions than GETs.

### Hypothesis 3

**H3a:** Teachers who remember that a fictitious student is labeled with an SLN/LD diagnosis have lower performance expectations of the student than teachers who do not remember the diagnosis or who read the student’s description without a diagnostic label. These lower performance expectations manifest in the intention to reduce the student’s academic requirements.
**H3b:** SETs have lower performance expectations of the fictitious student than GETs. These lower performance expectations manifest in the intention to reduce the student’s academic requirements.

## Method

### Procedure

Our study was conducted online with LimeSurvey (2021). Teachers were randomly assigned to the control (no label) or one of two experimental groups (LD or SLN label). In all groups, they read the same vignette describing a fictitious student with learning problems. Only in the experimental groups was the fictitious student labeled with either an LD or SLN diagnosis in addition to the description of the learning problems (see section “Vignette”). Teachers then answered three simple content questions to check for careful reading. After teachers assessed the dependent variables, the study finished with a manipulation check and the collection of demographic information. Participants received 40 € for their participation.

### Participants

Our participants were in-service GETs and SETs in North Rhine Westphalia, Germany. The questionnaire was started 543 times. Fifty-one teachers started or conducted the questionnaire more than once. We only kept the first participation of each teacher, which resulted in the removal of 63 participations (11.60%), leaving 480 participants. We excluded 28 teachers (5.8%) because they had not read the vignette carefully. They gave no or wrong answers to more than one of the three comprehension questions to check for careful reading. A further 22 participants (4.87%) were excluded because they did not indicate their profession, and four participants (0.93%) were neither a GET nor a SET. As in our previous studies (Kashikar et al., 2023, 2024) and thus expected, some teachers of the two experimental groups (SLN or LD diagnosis presented in the fictitious student’s description) incorrectly reported that the respective diagnosis was not mentioned in the vignette (see Note 2). Although we did not hypothesize that the labels unconsciously influenced teachers’ performance expectations, we did not exclude these teachers. Instead, we set up a separate experimental group to examine whether the diagnoses even influenced teachers’ judgments when not consciously remembered. We thus adapted the one control group (CG) and the two experimental groups to four different label groups (LGs). LG0 contained teachers who read the fictitious student’s description with no diagnosis and who correctly did not remember a diagnosis being mentioned. LG1 comprised teachers who read the vignette with the SLN or LD diagnosis but incorrectly did not remember that a diagnosis was mentioned. LG2 and LG3 consisted of teachers who read the vignette with the SLN (LG2) or LD (LG3) diagnosis and correctly remembered that the diagnosis was mentioned (see Table 1). This procedure resulted in the exclusion of 13 (3.05%) teachers of the former CG (vignette without diagnosis), who incorrectly stated that the SLN or LD label had been presented in the student’s description, as the group was too small to form another LG. No outliers were excluded.

**Table 1.** Distribution of General and Special Education Teachers in the Label Groups.

Description	LG0	LG1	LG2	LG3	Total
	No diagnosis was presented or remembered.	SLN or LD diagnosis was presented but not remembered.	SLN diagnosis was presented and remembered.	LD diagnosis was presented and remembered.	
GETs	76	56	60	53	245
SETs	53	39	34	42	168
Total	129	95	94	95	413

*Note.* LG = label group; GETs = general education teachers; SETs = special education teachers. The new label groups were formed by the manipulation check: Teachers were asked to remember the vignette they had read in the beginning and to answer one of the following questions, which depended on the vignette they read: Was it mentioned in the description that L. has a learning disability or special learning needs? (no diagnosis in the student’s description)/Was it mentioned in the description that L. has special learning needs? (SLN diagnosis in the student’s description)/Was it mentioned in the description that L. has a learning disability? (LD diagnosis in the student’s description). The question had to be answered with *yes* or *no*.

The final sample included 413 teachers with an average age of 41.01 (standard deviation [*SD*] = 11.01) years. In the sample, there were 79.18% female teachers, 20.10% male teachers, and 0.01% were diverse or did not indicate their gender. At the time of data collection, 64.16% of the teachers worked in general schools, and the remaining 35.84% worked in special schools. Of the teachers in general education, 64.52% worked in inclusive schools. On average, teachers had 12.96 (*SD* = 9.83) years of professional experience. The distribution of GETs and SETs did not differ between the four LGs, χ^2^ = 1.30, *df* = 3, *p* = .730, Cramer’s *V* = .056.

### Measures

#### Vignette

The student description was adapted from Lanfranchi and Jenny (2005) and read in the initial CG and LGs as presented below. In the LGs, teachers were presented with either the SLN or the LD diagnosis, not with both diagnoses:

L. is ten years old (*and has Special Learning Needs/and has a Learning Disability*). Since the first grade, L. has shown learning and performance problems in reading, writing, and mathematics, which have increased over time. L. works very slowly, is quickly overwhelmed, and appears helpless. It is difficult for L. to retain learned facts over a longer period. When calculating, L. often uses fingers. Moreover, L. reads familiar words very slowly and makes many spelling mistakes when writing.

The instruction was also different in the CG and LGs: “You should now work on a few tasks. In this context, imagine you are the class teacher in the class of *(the student with Special Learning Needs/the learning-disabled student)* L.”

#### Teachers’ Beliefs on Whether the SLN/LD Diagnosis Is Correct

Depending on which vignette they read, teachers judged whether the fictitious student had SLN or an LD (vignette without label), SLN (vignette with SLN label), or an LD (vignette with LD label). The question had to be answered with *yes* or *no*.

#### Stereotypes

##### Stereotype Content Model

Teachers provided ratings of the fictitious students’ *warmth* and *competence* using the scales of Fiske et al. (2002). Warmth and competence items were presented together in randomized order. Warmth was assessed by ratings of how well the adjectives “warm,” “tolerant,” “sincere,” and “good-natured” described the fictitious student (α = .88). Competence was assessed by ratings of how well the adjectives “intelligent,” “independent,” “competent,” and “confident” described the fictitious student (α = .76). All items were assessed on a 6-point Likert-type scale from 1, *does not apply at all*, to 6, *fully applies*.

##### Paternalistic Emotions

Teachers rated how much *pity* and *sympathy* they felt for the fictitious student on a 6-point Likert-type scale from 1, *none*, to 6, *a lot*, for pity and 1, *very unlikable*, to 6, *very likable*, for sympathy.

#### Performance Expectations

##### Likelihood of a Poor Grade in the Next Class Test

Teachers were asked to indicate the likelihood that the fictitious student would score a bad grade in the next class assignment on a 6-point Likert-type scale from 1, *very unlikely*, to 6, *very likely*.

##### Likelihood of Achieving the Class Target

Teachers were asked to indicate the likelihood that the fictitious student would reach the curriculum class target for the current school year on a 6-point Likert-type scale from 1, *very unlikely*, to 6, *very likely*.

##### Highest Expected Graduation Level

Teachers were asked to estimate the highest graduation level the fictitious student could achieve. The item was assessed on a 6-point Likert-type scale, with 1 indicating *no graduation*, 2 indicating the *graduation level that children who have a special education diagnosis in the category learning can legally achieve*, 3 representing the *basic track graduation*, 4 representing the *intermediate track graduation*, 5 indicating the *lower academic track graduation*, and 6 meaning the *highest academic track graduation*.

##### Likelihood of Completing Vocational Training

Teachers indicated the likelihood that the fictitious student would complete vocational training on a 6-point Likert-type scale from 1, *very unlikely*, to 6, *very likely*.

##### Likelihood of Completing a University Degree

Teachers were asked to indicate the likelihood that the fictitious student will graduate from university on a 6-point Likert-type scale from 1, *very unlikely*, to 6, *very likely*.

##### Likelihood of Working in the First Labor Market

In Germany, the first labor market is free and distinguished from the second labor market, which is the labor market for people with disabilities. Teachers rated the likelihood that the fictitious student would work in the first labor market on a 6-point Likert-type scale from 1, *very unlikely*, to 6, *very likely*.

#### Manifestation of Teachers’ Expectations in Behavioral Intentions

##### Choice of Math Tasks

Teachers were presented with 15 blocks of math tasks, each containing five multiplication tasks per block. They were instructed to select all blocks; they thought the fictitious student could solve successfully within 5 min. The task blocks varied in difficulty, with five blocks of easy tasks (e.g., 1 × 2, 10 × 5, presented in green), five blocks of medium tasks (e.g., 2 × 4, 3 × 3, presented in yellow), and five blocks of complex tasks (e.g., 3 × 7, 6 × 9, presented in red; Buchwald et al., 2022). A sum was calculated to examine the task choice: One point was given for each easy task block selected, two points for each medium task block, and three points for each complex task block. Thus, the point range was between 0 and 30 points.

##### Intention to Foster Performance

We developed four items to measure the extent to which teachers would intend to adapt their teaching to foster the academic performance of the fictitious student (e.g., “I would adapt my teaching to ensure that L. develops a high-performance motivation” and “I would adapt my teaching to ensure that L. achieves good grades in examinations”). The items were measured on a 6-point Likert-type scale from 1, *little adaption*, to 6, *strong adaption* (α = .66).

##### Track Recommendation for Secondary School

For the transition to a secondary school, teachers provide a (in some federal states binding) track recommendation at the end of primary school in Germany. The general secondary school system has traditionally been three-tiered, consisting of *basic*, *intermediate*, and *academic track schools*. Comprehensive schools have been growing and replacing basic and intermediate track schools in recent years. Most comprehensive schools also offer academic track education and thus combine all secondary education levels. Besides the general education system, special schools for students with disabilities exist. In our study, participants were informed that as class teachers, they have to give a track recommendation for the fictitious student’s secondary school. The choice was between *special*, *basic*, *intermediate*, *academic*, or *comprehensive school.*

### Statistical Analyses

Analyses of variance (ANOVAs) using Type III sum of squares were conducted for all dependent variables except for the stereotypical perception of whether the fictitious student has an SLN/LD and the track recommendation. For these two variables, we carried out binomial logistic regressions. This was necessary for the track recommendation because although educational tracks in Germany can generally be ranked hierarchically according to their academic requirements, comprehensive schools cannot be positioned anywhere in this order, as they offer all graduation levels. Independent variables/predictors for all analyses was the LG (LG0 vs. LG1 vs. LG2 vs. LG3), the teaching profession (GETs vs. SETs), and their interaction. In the case of post hoc tests, we used the Tukey method to adjust the *p* values for multiple tests. We used pairwise deletion for analyses because of the very low percentage of missing data in the dependent variables (0.33%). Analyses were conducted with R 4.3.2 (R Core Team, 2023) and RStudio 2023.12.0 (RStudio Team, 2023). We used *afex* 1.0-1 (Singmann et al., 2023), *tidyverse* 1.2.1 (Wickham et al., 2019), and *WRS2* (Mair & Wilcox, 2020).

## Results

### Descriptive Results and Correlations

Table 2 displays the frequencies of teachers’ beliefs on whether the SLN/LD diagnosis is correct. Table 3 shows the results of the paternalistic stereotypes and emotions. Table 4 displays the results of the performance expectations; Tables 5 and 6 show their manifestation in behavioral intentions. The intercorrelations of the study variables are displayed in Table 7.

**Table 2.** Frequency Table of the Stereotypical Perception of Whether the Fictitious Student Has Special Learning Needs/a Learning Disability.

Profession	Assessment	LG0	LG1	LG2	LG3
GETs	No	19	10	6	5
	Yes	57	46	54	48
SETs	No	15	10	4	2
	Yes	38	29	30	40

*Note.* The questions varied according to the vignette teachers read. Teachers who read the vignette without diagnosis judged whether the fictitious student had SLN or an LD. Teachers who read the vignette with the SLN diagnosis judged whether the fictitious student had SLN, and teachers who read the vignette with the LD diagnosis judged whether the fictitious student had an LD. LG = label group; GETs = general education teachers; SETs = special education teachers; SLN = special learning needs.

**Table 3.** Means and Standard Deviations of the Paternalistic Stereotypes and Emotions.

		CG		LG1		LG2		LG3		Total	
Variables	Teaching Profession	*M*	*SD*	*M*	*SD*	*M*	*SD*	*M*	*SD*	*M*	*SD*
Stereotype—warmth	GETs	4.10	0.79	4.22	0.90	4.23	0.94	4.22	0.81	4.19	0.86
	SETs	4.29	0.82	4.23	0.81	4.31	0.93	4.14	0.87	4.24	0.85
	Total	4.18	0.80	4.22	0.86	4.26	0.93	4.18	0.83	4.21	0.85
Stereotype—competence	GETs	2.88	0.68	2.97	0.73	2.90	0.88	3.05	0.82	2.94	0.77
	SETs	3.02	0.80	3.11	0.98	3.03	0.77	2.95	0.65	3.02	0.80
	Total	2.94	0.73	3.03	0.84	2.95	0.84	3.01	0.75	2.98	0.78
Pity	GETs	4.26	1.25	3.84	1.41	4.35	1.33	3.92	1.40	4.11	1.34
	SETs	3.34	1.41	3.97	1.33	3.59	1.56	3.40	1.48	3.55	1.45
	Total	3.88	1.39	3.89	1.37	4.07	1.45	3.69	1.45	3.89	1.42
Sympathy	GETs	4.32	0.79	4.42	0.90	4.52	0.91	4.45	0.91	4.42	0.87
	SETs	4.66	1.06	4.54	0.91	5.03	0.81	4.55	1.19	4.68	1.03
	Total	4.46	0.92	4.47	0.90	4.70	0.91	4.49	1.04	4.52	0.94

*Note*. CG = control group; LG = label group; GETs = general education teachers; SETs = special education teachers.

**Table 4.** Means and Standard Deviations of the Performance Expectations.

		LG0		LG1		LG2		LG3		Total	
Variable	Teaching Profession	*M*	*SD*	*M*	*SD*	*M*	*SD*	*M*	*SD*	*M*	*SD*
Likelihood of a poor grade in the next class test	GETs	3.73	0.95	3.57	0.91	3.67	1.10	3.87	1.37	3.71	1.08
	SETs	3.77	1.02	3.61	0.89	3.41	1.21	3.67	1.10	3.63	1.05
	Total	3.75	0.98	3.59	0.90	3.57	1.14	3.78	1.25	3.68	1.07
Likelihood of achieving the class target	GETs	2.83	1.11	2.95	1.07	2.72	1.17	2.36	1.00	2.73	1.11
	SETs	2.92	1.05	2.59	1.09	2.68	1.20	2.79	1.07	2.76	1.10
	Total	2.87	1.09	2.80	1.09	2.70	1.17	2.55	1.05	2.74	1.10
Highest expected graduation level	GETs	3.67	0.93	3.41	1.01	3.48	1.16	3.26	0.76	3.48	0.98
	SETs	3.23	0.85	3.03	0.96	2.82	0.90	2.69	0.64	2.96	0.86
	Total	3.49	0.92	3.25	1.00	3.24	1.11	3.01	0.76	3.27	0.97
Likelihood of completing vocational training	GETs	4.49	1.09	4.38	1.20	4.57	1.09	4.21	1.21	4.42	1.14
	SETs	4.21	1.08	3.79	1.03	3.65	1.39	3.74	1.25	3.88	1.19
	Total	4.37	1.09	4.14	1.16	4.23	1.28	4.00	1.25	4.20	1.19
Likelihood of completing a university degree	GETs	2.11	1.16	1.89	1.02	1.88	1.12	1.75	1.22	1.93	1.14
	SETs	1.96	1.14	1.85	1.25	1.68	0.91	1.33	0.61	1.72	1.04
	Total	2.05	1.15	1.87	1.11	1.81	1.05	1.57	1.02	1.84	1.10
Likelihood of working in the first labor market	GETs	4.28	1.10	4.21	1.04	4.07	1.06	3.96	1.30	4.14	1.12
	SETs	4.21	1.17	3.79	1.08	3.74	1.58	3.98	1.20	3.96	1.25
	Total	4.25	1.13	4.04	1.07	3.95	1.27	3.97	1.25	4.07	1.18

*Note*. LG = label group; GETs = general education teachers; SETs = special education teachers.

**Table 5.** Means and Standard Deviations of the Manifestation of Teachers’ Expectations in Behavioral Intentions.

		LG0		LG1		LG2		LG3		Total	
Variable	Teaching Profession	*M*	*SD*	*M*	*SD*	*M*	*SD*	*M*	*SD*	*M*	*SD*
Choice of math tasks	GETs	6.75	3.78	7.59	3.73	6.63	3.49	7.47	4.45	7.07	3.85
	SETs	6.60	3.03	6.44	3.55	6.65	3.58	6.38	4.14	6.52	3.53
	Total	6.69	3.48	7.12	3.68	6.64	3.50	6.99	4.33	6.85	3.73
Intention to foster performance	GETs	3.74	0.67	3.79	0.73	3.85	0.77	3.69	0.88	3.77	0.75
	SETs	3.89	0.88	4.01	0.72	4.23	0.80	3.73	0.86	3.95	0.84
	Total	3.80	0.77	3.88	0.73	3.99	0.80	3.71	0.86	3.84	0.79

*Note*. LG = label group; GETs = general education teachers; SETs = special education teachers.

**Table 6.** Frequency Table of the Track Recommendation to Special School versus a General School.

Teaching Profession	Recommendation	LG0	LG1	LG2	LG3
GETs	Special school	14	13	17	18
	General school	62	43	42	35
SETs	Special school	21	16	17	29
	General school	32	23	17	13

*Note*. LG = label group; GETs = general education teachers; SETs = special education teachers.

**Table 7.** Correlations for the Dependent Variables.

Variable	1	2	3	4	5	6	7	8	9	10	11	12	13	14
1 Acceptance of diagnosis	—													
2 Stereotype warmth	−.02	—												
3 Stereotype competence	−.14**	.40**	—											
4 Pity	−.03	.11*	−.13**	—										
5 Sympathy	.01	.32**	.18**	.12*	—									
6 Poor grade	.14**	−.09	−.23**	.19**	−.08	—								
7 Class target	−.29**	.08	.18**	−.14**	.04	−.33**	—							
8 Graduation level	−.30**	.08	.30**	.02	.01	−.20**	.29**	—						
9 Vocational training	−.20**	.24**	.32**	.03	.14**	−.21**	.23**	.43**	—					
10 University degree	−.26**	.03	.33**	−.05	.00	−.22**	.21**	.54**	.26**	—				
11 First labor market	−.15**	.22**	.28**	−.05	.12*	−.16**	.22**	.30**	.65**	.27**	—			
12 Track recommendation	−.26**	.05	.10*	−.01	.01	−.12*	.25**	.48**	.29**	.19**	.21**	—		
13 Choice of math tasks	−.05	.05	.16**	−.01	.01	−.11*	.14**	.15**	.09	.14**	.06	.12*	—	
14 Fostering of performance	−.06	.16**	.12*	.05	.09	−.17**	.13**	.12*	.14**	.07	.13**	−.01	.08	—

*Note*. Track recommendation coded with 0 = special school, 1 = any type of general school. Acceptance of diagnosis: Do you think L. has special learning needs/a learning disability? No (0), yes (1).

* *p* < .05. **p < .01.

### Hypotheses Testing

#### Teachers’ Beliefs on Whether the SLN/LD Diagnosis Is Correct

The binomial logistic regression model was significant. With the LG0 and GETs set as the respective reference groups, LG2 (i.e., teachers who remembered the SLN label), *p* = .030, and LG3 (i.e., teachers who remembered the LD label), *p* = .031, were significant predictors for believing in the presence of the respective diagnosis. Specifically, in LG0, 73.64% thought the fictitious student had SLN or an LD. In LG2, 89.36% of the teachers assumed the presence of SLN, and 92.63% of the teachers in LG3 assumed the presence of an LD. No other predictors were significant (see Table 8).

**Table 8.** Result of a Logistic Regression Examining Whether the Label Group and Teaching Profession Influence the Teachers’ Belief on Whether the SLN/LD Diagnosis Is Correct.

Variable	*B*	*SE*	*z*	Exp (*B*)	*p*	95% CI
Intercept	1.10	0.26	4.15	3.00	<.001	[0.59, 1.64]
LG1	0.43	0.44	0.98	1.53	.329	[−0.41, 1.32]
LG2	1.10	0.51	2.17	3.00	.030	[0.16, 2.17]
LG3	1.16	0.54	2.16	3.20	.031	[0.17, 2.33]
SETs	−0.17	0.40	−0.42	0.84	.676	[−0.96, 0.63]
LG1 × SETs	−0.29	0.65	−0.45	0.75	.652	[−1.57, 0.98]
LG2 × SETs	−0.01	0.79	−0.02	0.99	.987	[−1.56, 1.61]
LG3 × SETs	0.90	0.95	0.95	2.47	.344	[−0.88, 3.01]

*Note.* Reference groups: LG0 and GETs. Do you think L. has special learning needs/a learning disability? No (0) versus yes (1). The binomial logistic regression model was significant, χ^2^(7) = 20.63, *p* =.004, Nagelkerke *R*^2^ = .081. SLN = special learning needs; LG = label group; SETs = special education teachers; GETs = general education teachers.

#### Stereotypes

##### Stereotype Warmth

There was no significant effect of the LG, $\eta_{p}^{2}$ = .001, the teaching profession, $\eta_{p}^{2}$ = .001, and their interaction, $\eta_{p}^{2}$ = .004 (see Table 9).

**Table 9.** Results of ANOVAs Examining Whether the Label Group and Teaching Profession Influence Teachers’ Stereotypes and Emotions.

Variable	Sum of squares	*df*	*F*	*p*	_partial_ η^2^	_partial_ η^2^ 95% CI [LL, UL]
Stereotype warmth						
Intercept	6,821.9	1	9,304.99	<.001		
Label group	0.4	3	0.19	.905	.001	[.000, .007]
Teaching profession	0.2	1	0.32	.569	.001	[.000, .015]
Group × profession	1.1	3	0.48	.696	.004	[.000, .016]
Error	291.8	398				
Stereotype competence						
Intercept	3,457.0	1	5,587	<.001		
Label group	0.5	3	0.25	.859	.002	[.000, .010]
Teaching profession	0.5	1	0.89	.347	.002	[.000, .020]
Group × profession	1.0	3	0.56	.639	.004	[.000, .018]
Error	248.1	401				
Pity						
Intercept	5,731.1	1	3,003.28	<.001		
Label group	4.9	3	0.85	.465	.006	[.000, .023]
Teaching profession	26.1	1	13.67	<.001	.033	[.007, .072]
Group × profession	16.1	3	2.82	.039	.020	[.000, .049]
Error	772.9	405				
Sympathy						
Intercept	8,034.7	1	9,208.12	<.001		
Label group	5.4	3	2.06	.104	.015	[.000, .040]
Teaching profession	6.9	1	7.90	.005	.019	[.002, .053]
Group × profession	2.6	3	1.01	.387	.007	[.000, .026]
Error	350.8	402				

##### Stereotype Competence

We found no significant effect of the LG, $\eta_{p}^{2}$ = .002, the teaching profession, $\eta_{p}^{2}$ = .002, and their interaction, $\eta_{p}^{2}$ = .004 (see Table 9).

##### Pity

The analysis indicated a main effect of the teaching profession, $\eta_{p}^{2}$ = .033. GETs felt more pity for the fictitious student than SETs. The significant interaction, $\eta_{p}^{2}$ = .020, indicated that the difference between GETs and SETs was significant in LG0, *p* < .001, and LG2, *p* = .011. We found no significant main effect of the LG, $\eta_{p}^{2}$ = .006 (see Table 9).

##### Sympathy

A main effect of the teaching profession, $\eta_{p}^{2}$ = .019, showed that SETs felt more sympathy for the fictitious student than GETs. The LG’s effect, $\eta_{p}^{2}$ = .015, and the interaction, $\eta_{p}^{2}$ = .007, were insignificant (see Table 9).

#### Performance Expectations

##### Likelihood of a Poor Grade in the Next Class Test

The Levene test showed that the assumption of variance homogeneity was violated, *F*(7,401) = 2.78, *p* = .008. We, therefore, conducted a robust ANOVA. Instead of the *F* distribution, the pairwise trimmed mean difference psi hat ( $\hat{\psi }$ ) was evaluated (Mair & Wilcox, 2020). We found no significant main effect of the teaching profession, $\hat{\psi }$ = 1.30, *p* = .256, the LG, $\hat{\psi }$ = 2.46, *p* = .491, or their interaction, $\hat{\psi }$ = 3.00, *p* = .401.

##### Likelihood of Achieving the Class Target

We found no significant effect of the teaching profession, $\eta_{p}^{2}$ = .000, the LG, $\eta_{p}^{2}$ = .011, or their interaction, $\eta_{p}^{2}$ = .015 (see Table 10).

**Table 10.** Results of ANOVAs Examining Whether the Label Group and Teaching Profession Influence Teachers’ Classic Performance Expectation.

Variable	Sum of squares	*df*	*F*	*p*	_partial_ η^2^	_partial_ η^2^ 95% CI [LL, UL]
Likelihood of achieving the class target						
Intercept	2,900.0	1	2,418.52	<.001		
Label group	5.2	3	1.45	.228	.011	[.000, .032]
Teaching profession	0.1	1	0.08	.777	.000	[.000, .011]
Group × profession	7.4	3	2.05	.107	.015	[.000, .040]
Error	485.6	405				
Highest expected graduation level						
Intercept	3,987.8	1	4,670.67	<.001		
Label group	12.4	3	4.86	.002	.035	[.005, .070]
Teaching profession	25.9	1	30.35	<.001	.070	[.029, .120]
Group × profession	1.1	3	0.42	.739	.003	[.000, .014]
Error	345.7	405				
Likelihood of completing vocational training						
Intercept	6,638.4	1	4,937.48	<.001		
Label group	8.3	3	2.07	.104	.015	[.000, .040]
Teaching profession	30.8	1	22.89	<.001	.053	[.019, .100]
Group × profession	5.4	3	1.34	.261	.010	[.000, .030]
Error	544.5	405				
Likelihood of completing a university degree						
Intercept	1,271.8	1	1,072.43	<.001		
Label group	13.2	3	3.71	.012	.027	[.001, .058]
Teaching profession	4.1	1	3.43	.064	.008	[.000, .034]
Group × profession	1.8	3	0.50	.680	.004	[.000, .016]
Error	480.3	405				
Likelihood of working in the first labor market						
Intercept	6,324.5	1	4,566.22	<.001		
Label group	7.3	3	1.76	.153	.013	[.000, .036]
Teaching profession	4.0	1	2.85	.092	.007	[.000, .031]
Group × profession	3.1	3	0.74	.528	.005	[.000, .021]
Error	561.0	405				

##### Highest Expected Graduation Level

We found a main effect of the LG, $\eta_{p}^{2}$ = .035, and the teaching profession, $\eta_{p}^{2}$ = .065. GETs expected a higher graduation level than SETs. The variables did not interact, $\eta_{p}^{2}$ = .003. The post hoc test for the effect of the LG showed a significant difference between LG0 and LG3, *p* = .001. This means teachers who remembered the LD label expected a lower graduation level than teachers who read the vignette without a label (see Table 10).

##### Likelihood of Completing Vocational Training

We found a main effect of the teaching profession, $\eta_{p}^{2}$ = .053. GETs rated the likelihood higher than SETs. We observed no significant effect of the LG, $\eta_{p}^{2}$ = .015, or interaction, $\eta_{p}^{2}$ = .010 (see Table 10).

##### Likelihood of Completing a University Degree

We found a main effect of the LG, $\eta_{p}^{2}$ = .027. The post hoc test indicated that only the difference between LG0 and LG3 was significant, *p* = .006. Teachers who remembered the LD label perceived a lower likelihood that the fictitious student would graduate from university than teachers who read the vignette without a label. We found no significant effect of the teaching profession, $\eta_{p}^{2}$ = .008, or interaction, $\eta_{p}^{2}$ = .004 (see Table 10).

##### Likelihood of Working in the First Labor Market

We found no significant effects of the LG, $\eta_{p}^{2}$ = .013, the teaching profession, $\eta_{p}^{2}$ = .007, or their interaction, $\eta_{p}^{2}$ = .005 (see Table 10).

#### Manifestation of Teachers’ Expectations in Behavioral Intentions

##### Choice of Math Tasks

There were no significant effects of the LG, $\eta_{p}^{2}$ = .002, the teaching profession, $\eta_{p}^{2}$ = .006, or their interaction, $\eta_{p}^{2}$ = .005 (see Table 11).

**Table 11.** Results of ANOVAs Examining Whether the Label Group and Teaching Profession Influence the Manifestation of Teachers’ Expectations in Behavioral Intentions.

Variable	Sum of squares	*df*	*F*	*p*	_partial_ η^2^	_partial_ η^2^ 95% CI [LL, UL]
Choice of math tasks						
Intercept	18,088.4	1	1,292.74	<.001		
Label group	9.8	3	0.23	.874	.002	[.000, .009]
Teaching profession	34.4	1	2.46	.118	.006	[.000, .029]
Group × profession	27.2	3	0.65	.585	.005	[.000, .019]
Error	5,666.9	405				
Intention to foster performance						
Intercept	5,825.3	1	9,451.35	<.001		
Label group	5.3	3	2.88	.036	.021	[.000, .049]
Teaching profession	3.7	1	6.04	.014	.015	[.000, .045]
Group × profession	1.4	3	0.76	.519	.006	[.000, .021]
Error	249.6	405				

##### Intention to Foster Performance

We found a significant effect of the LG, $\eta_{p}^{2}$ = .021. The post hoc test showed a significant difference between LG2 and LG3, *p* = .025. This means teachers who remembered the SLN label intended to foster performance more than teachers who remembered the LD label. The teaching profession’s main effect was also significant, $\eta_{p}^{2}$ = .015. SETs intended to foster performance more than GETs. There was no significant interaction, $\eta_{p}^{2}$ = .006 (see Table 11).

##### Track Recommendation for Secondary School

We tested the frequency of recommendations for a special school compared with any general school. The binomial logistic regression model was significant. With the LG0 and GETs set as the respective reference groups, LG3, *p* = .047, was a significant predictor for recommending a special school. Specifically, in LG0, only 27.13% recommended a special school, but 49.47% of teachers who remembered the LD label recommended a special school. Being an SET also predicted recommendations to a special school, *p* = .009. Specifically, 49.40% of the SETs advised a special school compared with 25.41% of the GETs. There were no significant interactions (see Table 12).

**Table 12.** Result of a Logistic Regressions Examining Whether the Label Group and Teaching Profession Influence the Track Recommendation.

Variable	*B*	*SE*	*z*	Exp (*B*)	*p*	95% CI
Intercept	1.49	0.30	5.03	4.43	<.001	[0.94, 2.11]
LG1	−0.29	0.43	−0.67	0.75	.501	[−1.15, 0.57]
LG2	−0.58	0.41	−1.42	0.56	.157	[−1.40, 0.22]
LG3	−0.82	0.41	−1.99	0.44	.047	[−1.65, −0.02]
SETs	−1.07	0.41	−2.62	0.34	.009	[−1.88, −0.28]
LG1 × SETs	0.23	0.61	0.38	1.26	.702	[−0.97, 1.43]
LG2 × SETs	0.16	0.61	0.27	1.18	.789	[−1.03, 1.35]
LG3 × SETs	−0.40	0.60	−0.67	0.67	.506	[−1.59, 0.78]

*Note.* Reference groups: LG0 and GETs. Track recommendation: special school (0) versus any type of general school (1). The binomial logistic regression model was significant, χ^2^(7) = 39.32, *p* <.001, Nagelkerke *R*^2^ = .125. LG = label group; SETs = special education teachers.

## Discussion

### Summary of the Results

Our study examined whether the two special education needs labels “SLNs” and “LD” influenced teachers’ judgments of a fictitious student with learning problems. We also examined whether the labels influenced GETs and SETs differently.

For our RQ1, we hypothesized that teachers who remembered the SLN/LD diagnosis believed more frequently that the diagnosis was correct (H1a) and that SETs did so more frequently than GETs (H1b). Hypothesis 1a was confirmed because teachers who remembered the SLN and LD labels were more likely to believe that the diagnosis was accurate. However, the teaching profession was not a significant predictor (H1b).

For the RQ2, we hypothesized that teachers who remembered the SLN/LD diagnosis had higher paternalistic stereotypes and emotions for the student than teachers who did not remember the diagnosis or read the student’s description without a diagnostic label (H2a). We also assumed SETs have higher paternalistic stereotypes and emotions than GETs (H2b). However, the SLN and LD labels did not significantly alter teachers’ perceived warmth or competence. The results regarding the influence of the teaching profession were more complex. As hypothesized, SETs felt more sympathy for the fictitious students. However, GETs felt more pity than SETs when no label was presented or they remembered the SLN label, which contradicts our assumption (discussed below).

For the RQ3, we hypothesized that teachers who remembered the SLN/LD diagnosis in the student’s description had lower performance expectations than teachers who did not remember or were not presented with a diagnosis. We also assumed that the lower performance expectations manifested in the intention to reduce students’ academic requirements (H3a). Our data partially supported our hypothesis because teachers who remembered the LD label expressed lower long-term academic expectations. These lower expectations manifested in more recommendations to a special school. Unexpectedly, we found that teachers who remembered the SLN label intended to foster the academic performance of the fictitious student more than teachers who remembered the LD label (discussed below).

We also hypothesized that SETs have lower performance expectations than GETs, which manifest in lower teaching requirements, regardless of the presence of a label (H3b). Our data supported this assumption somewhat because some of the SETs’ expectations were lower than those of GETs. However, despite some lower expectations, SETs intended to foster academic performance more than GETs, contradicting the assumed connections between expectations and their manifestation in behavioral intentions.

### Interpretation of the Results and Outlook on Future Research

Like in other studies, the diagnostic labels increased the likelihood that teachers believed the diagnosis was accurate (Kashikar et al., 2023, 2024; Shifrer, 2013). However, unlike postulated by the label theory, the labels did not increase paternalistic stereotypes, which was previously observed for students with learning problems (Krischler & Pit-ten Cate, 2019). Two interconnected explanations for this result could be considered. First, we measured teachers’ explicit stereotypes; meaning teachers were aware of their judgments (Greenwald & Banaji, 1995). It is possible that socially desirable responses influenced the results obtained. Although teachers perceived the student’s warmth to be higher than the competence, typically associated with individuals with disabilities, the actual competence score was around the average point on the scale and not as low as predicted by the stereotype content model (Fiske et al., 2002). Thus, teachers may have given socially desirable answers. Second, implicit bias needs to be considered. Unlike explicit measures, “implicit bias is thought to reflect the automatic cognitive association or affective predisposition individuals have with different social groups” (Starck et al., 2020, p. 2). Individuals are believed to have limited awareness and control over their implicit biases (Greenwald & Banaji, 1995). Teachers’ implicit biases have primarily been studied for Caucasian versus non-Caucasian students (Chin et al., 2020; Kumar et al., 2015; Starck et al., 2020). However, teachers may also have implicit biases toward students with special educational needs. Although implicit beliefs can be challenging to measure and may not manifest in behavior (Lavigne & Good, 2021), future studies should examine teachers’ explicit stereotypes and implicit bias of students with learning problems to achieve a more holistic view.

As mentioned, the competence and warmth dimensions were higher and lower than anticipated by the stereotype content model. A caveat that needs to be considered is the meaning of “competence” and “warmth” in teacher–student relationships. Although the stereotype content model has been used to evaluate group stereotypes in many contexts (Gärtner et al., 2022; Vervecken et al., 2015), it has to be considered that a group’s perceived competition conceptualizes the meaning of warmth, and a group’s perceived status conceptualizes the meaning of competence (Cuddy et al., 2007; Fiske et al., 2002). This means that groups perceived as competitive are not perceived as warm, and vice versa. And, if the group’s status is perceived as high, it is perceived as competent, and vice versa. However, in the school context, there is a natural hierarchy where teachers have a higher status than students, and it is unlikely that teachers feel professional competition from students. This natural hierarchy might influence teachers’ ratings, and the question of what warmth measures from a teacher–student perspective arises. One possibility is that instead of competence in the classic sense, it measures teachers’ perceived self-efficacy to teach students with special educational needs in learning. Thus, when teachers feel unprepared to teach these students in inclusive settings (Wray et al., 2022), they might consider the needs of these students as a “threat” to teaching the curricular requirements.

One reason why teachers’ warmth and competence ratings were not as high or low for students with disabilities as suggested by the stereotype content model may be the difference between the overall stereotype of a group (like “students with SLN/an LD”) and the judgment of an individual student. Specifically, judging members of a social group might lead to more stereotypical assumptions than judgments of an individual who (possibly) is a member of a particular social group.

Even if this study did not indicate that the SLN/LD labels changed teachers’ stereotypes, we must remember that we examined the teachers’ perspectives. Although this perspective is critical, the stereotypes that labeled students perceive should not be neglected. Studies showed that labeled students perceived themselves as incompetent and thought others believed they were incompetent (May & Stone, 2010). They felt shame or humiliation and actively tried to conceal their disability to reduce negative stereotypes of teachers and peers (Daley & Rappolt-Schlichtmann, 2018). Thus, although one might argue that the results of this study do not strongly support the labeling-stereotype connection, it is likely that students labeled with learning disabilities still perceive stereotypes from their teachers, parents, peers, and other people in their environment.

Looking at paternalistic emotions and differences between the two teaching professions, we found that SETs feel more sympathy for the fictitious student than GETs. However, a study showed that solid sympathy toward disabled people predicted stable attributions of learning problems (Brady & Woolfson, 2008). This might help explain why SETs had lower performance expectations than GETs, which will be discussed later. Specifically, it might indicate that teachers’ sympathy for students is not closely related to performance expectations. More detailed, the intercorrelation table shows that sympathy was not related to performance expectations within the school context but to work-related expectations later in life.

The result that GETs felt more pity for the fictitious student in the absence of a label or when they remembered the SLN label is difficult to explain, especially because the effect did not occur for the LD label. Before we draw far-reaching conclusions, this result should be replicated.

As in previous studies, the LD label lowered teachers’ performance expectations when they remembered the diagnosis (Kashikar et al., 2023, 2024). However, the SLN label did not result in significantly lower teacher expectations. This contradicts the results of other studies that found adverse effects of different labels for students with learning problems (Hornstra et al., 2010). It seems that the SLN and LD labels influence teachers differently. A possible explanation could be found in the wording itself. An LD includes the word “disability,” which may evoke a medical perspective of an LD as inherent within the student (Ho, 2004). In contrast, the German translation of SLN emphasizes the educational need for support and may encourage the assumption that the student’s problems could decrease with the right help. This may explain why teachers who remembered the SLN label intended to foster the fictitious students’ performance more than teachers who remembered the LD label. Future studies should investigate the differential impact of the two diagnoses on other teacher judgments.

It is noteworthy that the LD label only lowered teachers’ long-term expectations. This might indicate that teachers perceived the diagnosis as stable. In other words, if the label only harmed short-term academic performance expectations, it would suggest that teachers were more likely to see learning problems as modifiable. This interpretation is reinforced because both labels led teachers to accept the respective diagnosis as correct more frequently.

The simplified expectation effect model postulates that teachers’ expectations manifest in their behavior (Johnston et al., 2019). Looking at the correlations between the expectation variables and the behavioral intention measures, our data supported the hypothesis that higher expectations manifested in higher requirements. However, SETs, who partially had lower expectations, intended to foster academic performance more than GETs. Thus, our results suggest that other factors, such as the teaching profession, may moderate the relation between expectations and behavior. Regardless of the SLN and LD labels, the present and our previous studies showed that SETs had lower performance expectations than GETs (Kashikar et al., 2023, 2024). However, as previously stated, other studies concluded the opposite (Vlachou et al., 2014; Woolfson et al., 2007). Maybe the type of school (i.e. a general or a special school) teachers work at influenced the lower performance expectations we found in our studies. Specifically, in this study, most SETs worked in special education (87.50%), compared with a small percentage of GETs (0.41%). The lower academic standards of special schools might have decreased SETs’ performance expectations. To examine this assumption, future studies should focus on the differences in the performance expectations of GETs and SETs depending on whether they teach in inclusive general or special schools.

### Limitations

Some limitations must be taken into consideration. First, our study was experimental, with a short vignette describing a fictitious student. Although such an approach is strong in internal validity, it typically lacks external validity. Likewise, measuring actual classroom behavior with a questionnaire is complex, such as the choice of tasks in experimental designs. Hence, labeling effects should be replicated in real-world classrooms. One possibility to measure teachers’ performance expectations is observing dyadic teacher–individual student interactions, as several teacher expectation effects studies have shown that teachers’ interaction with students can vary as a function of their perceived capability of the student (Lavigne & Good, 2021). Second, we demonstrated that the LD label only unfolded adverse effects on teachers’ judgments when consciously remembered. Future studies should investigate which teacher-related variables influence whether or not a label is perceived and remembered. Third, the group sizes of each LG were small. Thus, our data may lack the power to identify small labeling effects of some dependent variables. Fourth, although we know that the LD label affects teachers’ performance expectations negatively, we do not see how relatively strong the influence of the LD label is compared with other factors that influence teachers’ expectations. For example, the actual student performance might moderate the impact of the LD label on teachers’ expectations. In this study, the learning problems of the fictitious student were described as profound, possibly making it easy not to question an LD diagnosis. This assumption is supported by the result that when no diagnosis was given (i.e., in the CG), nearly three-quarters of the teachers believed in the diagnosis. However, can the effects of the LD label also be found when the described academic performance is less severe? This question should be investigated in further studies. Fifth, it has to be considered that the description of our fictitious student also did not inform teachers about gender, whether or not the student had a migration background, or their socio-economic status. We know that these variables, among others not mentioned, influence teachers’ judgments about students (for teachers’ performance expectations, see Wang et al., 2018). Hence, it is necessary to examine the possible interactions of these variables with the label effects in forthcoming studies.

## Conclusion

In the context of ableism in schools and the Dilemma of Difference, our study empirically examined the theoretical assumption that the use of diagnostic labels for students with learning problems is linked to the stereotype of low academic competence, which results in teachers’ lower performance expectations of this student. Our study showed that the “LD” label lowered teachers’ long-term academic performance expectations. However, our data did not support the assumption that this was caused by the label’s link to the stereotype of lower competence. Thus, more investigations and further studies measuring implicit bias are needed. If the label-stereotype/label-implicit bias connection was supported in forthcoming studies, it could be a starting point for teacher training to provide insights into the labeling effect. Our analysis also showed that different diagnostic labels for the same students lead to different teacher judgments. Although the development of diagnostic terms is carried out at a political level, it is essential to evaluate whether the changes in terminology lead to the intended changes in the perception of the diagnosed students.

## Notes

1. For readers interested in the underlying processes between the linking of labels and stereotypes: The social cognitive approach by Fiske (1998) examines the cognitive processes between the categorization of individuals (e.g., by a label) and the activation of stereotypes (Link & Phelan, 2001).

2. The fact that some teachers did not remember the diagnosis of SLN/an LD, although it was mentioned in the vignette, cannot be interpreted as inattentive reading, as they had correctly answered the three content questions that were asked immediately after reading the description of the fictitious student. Remember that the experimental manipulation was rather subtle and that the manipulation check as to whether the diagnosis was mentioned in the vignette was asked at the end of the questionnaire after around 15 min.
